# Supplementary material for: Pharmacological blood pressure control and outcomes in patients with hypertensive crisis discharged from the emergency department
Source: PLoS One. 2021 Aug 17;16(8):e0251311. doi: 10.1371/journal.pone.0251311 (PMC8370605; doi:10.1371/journal.pone.0251311)
Supplement: S1 Table — (DOCX) [file pone.0251311.s001.docx]

**S1 Table.** Type of antihypertensive drugs used for pharmacological control of blood pressure in the emergency department for hypertensive crisis (N= 6364).

| **Category** | **No. of Patients** | **%** |
| --- | --- | --- |
| Angiotensin- converting enzyme inhibitors /Angiotensin receptor blockers | 3240 | 50.9 |
| Calcium channel blockers | 3001 | 47.2 |
| Calcium channel blockers combination | 16 | 0.3 |
| Hydralazine | 72 | 1.1 |
| Others | 5 | 0.1 |
| Diuretics | 440 | 6.9 |
| Organic nitrates | 1212 | 19 |
| Potassium sparing diuretics | 35 | 0.5 |
| α/β blocker | 1319 | 20.7 |
