# Supplementary material for: Pharmacological blood pressure control and outcomes in patients with hypertensive crisis discharged from the emergency department
Source: PLoS One. 2021 Aug 17;16(8):e0251311. doi: 10.1371/journal.pone.0251311 (PMC8370605; doi:10.1371/journal.pone.0251311)
Supplement: S2 Table — (DOCX) [file pone.0251311.s002.docx]

**S2 Table.** The International Classification of Disease (ICD) codes and medications used to define comorbidities in this study.

| Comorbidities | ICD-9-CM |  | Medication |
| --- | --- | --- | --- |
| Diabetes | 250 | And | Insulin, Oral antidiabetic drugs |
| Hypertension | 401-405 | or | Angiotensin-converting-enzyme inhibitors (ACEIs), Angiotensin II receptor blockers (ARBs), Calcium channel blockers, Calcium channel blockers combination, Hydralazine, Diuretics, Organic nitrates, Potassium sparing diuretics, α/β blocker |
| Stroke | 430-438 |  | Not applicable |
| Cardiovascular disease | 410-414,428,441,442,429.2 |  | Not applicable |
