# Supplementary material for: Pharmacological blood pressure control and outcomes in patients with hypertensive crisis discharged from the emergency department
Source: PLoS One. 2021 Aug 17;16(8):e0251311. doi: 10.1371/journal.pone.0251311 (PMC8370605; doi:10.1371/journal.pone.0251311)
Supplement: S3 Table — (DOCX) [file pone.0251311.s003.docx]

**S3 Table.** Adjusted hazard ratios (HRs) and 95% confidence intervals of 7-day, 30-day, and 60-day ED revisit or inpatient admission by clinical characteristics of the study population.

|  | **Pharmacological BP intervention** | **Cases** | **N** | **Adjusted HR**  **(95% CI)^a^** |  | **Cases** | **N** | **Adjusted HR**  **(95% CI)^a^** | **P for interaction** |
| --- | --- | --- | --- | --- | --- | --- | --- | --- | --- |
| **Age < 65** |  |  |  |  | **Age ≥ 65** | | | |  |
| 7 days | No | 844 | 10522 | Reference |  | 595 | 6020 | Reference |  |
|  | Yes | 306 | 3540 | 0.99 (0.83 - 1.17) |  | 282 | 2824 | 0.9 (0.76 - 1.06) | 0.962 |
| 30 days | No | 1511 | 10522 | Reference |  | 1084 | 6020 | Reference |  |
|  | Yes | 518 | 3540 | 0.92 (0.81 - 1.05) |  | 506 | 2824 | 0.86 (0.76 - 0.98) | 0.556 |
| 60 days | No | 1872 | 10522 | Reference |  | 1395 | 6020 | Reference |  |
|  | Yes | 653 | 3540 | 0.92 (0.82 - 1.03) |  | 647 | 2824 | 0.86 (0.78 - 0.96) | 0.643 |
| **Female** |  |  |  |  | **Male** | | | |  |
| 7 days | No | 712 | 8793 | Reference |  | 727 | 7749 | Reference |  |
|  | Yes | 324 | 3597 | 0.95 (0.81 - 1.11) |  | 264 | 2767 | 0.94 (0.79 - 1.12) | 0.483 |
| 30 days | No | 1340 | 8793 | Reference |  | 1255 | 7749 | Reference |  |
|  | Yes | 565 | 3597 | 0.87 (0.77 - 0.98) |  | 459 | 2767 | 0.92 (0.81 - 1.05) | 0.997 |
| 60 days | No | 1679 | 8793 | Reference |  | 1588 | 7749 | Reference |  |
|  | Yes | 704 | 3597 | 0.85 (0.77 - 0.95) |  | 596 | 2767 | 0.94 (0.84 - 1.06) | 0.551 |
| **Non-diabetes** | | | | | **Diabetes** | | | |  |
| 7 days | No | 1230 | 14880 | Reference |  | 209 | 1662 | Reference |  |
|  | Yes | 473 | 5530 | 0.94 (0.82 - 1.07) |  | 115 | 834 | 1.01 (0.79 - 1.31) | 0.368 |
| 30 days | No | 2172 | 14880 | Reference |  | 423 | 1662 | Reference |  |
|  | Yes | 817 | 5530 | 0.9 (0.81 - 1) |  | 207 | 834 | 0.88 (0.73 - 1.06) | 0.7 |
| 60 days | No | 2715 | 14880 | Reference |  | 552 | 1662 | Reference |  |
|  | Yes | 1023 | 5530 | 0.89 (0.81 - 0.97) |  | 277 | 834 | 0.9 (0.77 - 1.06) | 0.39 |
| **Non-** **hypertension** | | | | | **Hypertension** | | | |  |
| 7 days | No | 862 | 11066 | Reference |  | 577 | 5476 | Reference |  |
|  | Yes | 121 | 1516 | 0.97 (0.75 - 1.26) |  | 467 | 4848 | 0.93 (0.81 - 1.06) | 0.933 |
| 30 days | No | 1480 | 11066 | Reference |  | 1115 | 5476 | Reference |  |
|  | Yes | 206 | 1516 | 1.01 (0.83 - 1.22) |  | 818 | 4848 | 0.86 (0.78 - 0.95) | 0.455 |
| 60 days | No | 1842 | 11066 | Reference |  | 1425 | 5476 | Reference |  |
|  | Yes | 250 | 1516 | 0.94 (0.79 - 1.13) |  | 1050 | 4848 | 0.87 (0.8 - 0.95) | 0.913 |
| **eGFR < 60** | | | | | **eGFR ≥ 60** | | | |  |
| 7 days | No | 400 | 2952 | Reference |  | 944 | 9623 | Reference |  |
|  | Yes | 214 | 1669 | 0.88 (0.72 - 1.07) |  | 356 | 3923 | 0.98 (0.85 - 1.14) | 0.736 |
| 30 days | No | 737 | 2952 | Reference |  | 1673 | 9623 | Reference |  |
|  | Yes | 392 | 1669 | 0.86 (0.75 - 1) |  | 600 | 3923 | 0.91 (0.81 - 1.02) | 0.979 |
| 60 days | No | 933 | 2952 | Reference |  | 2089 | 9623 | Reference |  |
|  | Yes | 505 | 1669 | 0.88 (0.78 - 1) |  | 754 | 3923 | 0.89 (0.81 - 0.99) | 0.848 |
| **Non-polypharmacy** | | | | | **Polypharmacy** | | | |  |
| 7 days | No | 903 | 11231 | Reference |  | 412 | 3130 | Reference |  |
|  | Yes | 370 | 4184 | 0.98 (0.85 - 1.14) |  | 169 | 1416 | 0.87 (0.71 - 1.06) | 0.12 |
| 30 days | No | 1577 | 11231 | Reference |  | 816 | 3130 | Reference |  |
|  | Yes | 619 | 4184 | 0.95 (0.84 - 1.06) |  | 323 | 1416 | 0.81 (0.71 - 0.94) | 0.039 |
| 60 days | No | 1949 | 11231 | Reference |  | 1045 | 3130 | Reference |  |
|  | Yes | 768 | 4184 | 0.93 (0.84 - 1.03) |  | 425 | 1416 | 0.83 (0.73 - 0.94) | 0.053 |
| **Non-survey of end-organ damage** | | | | | **Survey of end-organ damage** | | | |  |
| 7 days | No | 415 | 5948 | Reference |  | 1024 | 10594 | Reference |  |
|  | Yes | 83 | 1293 | 0.88 (0.65 - 1.19) |  | 505 | 5071 | 0.97 (0.85 - 1.11) | 0.141 |
| 30 days | No | 777 | 5948 | Reference |  | 1818 | 10594 | Reference |  |
|  | Yes | 136 | 1293 | 0.78 (0.61 - 0.98) |  | 888 | 5071 | 0.93 (0.84 - 1.02) | 0.004 |
| 60 days | No | 981 | 5948 | Reference |  | 2286 | 10594 | Reference |  |
|  | Yes | 176 | 1293 | 0.81 (0.65 - 0.99) |  | 1124 | 5071 | 0.92 (0.84 - 1) | 0.003 |
| **Second BP measurement not meeting HTN-C criteria** | | | | | **Second BP measurement also meeting HTN-C criteria** | | | |  |
| 7 days | No | 1260 | 14599 | Reference |  | 179 | 1943 | Reference |  |
|  | Yes | 313 | 3538 | 0.94 (0.82 - 1.09) |  | 275 | 2826 | 0.88 (0.7 - 1.11) | 0.901 |
| 30 days | No | 2290 | 14599 | Reference |  | 305 | 1943 | Reference |  |
|  | Yes | 551 | 3538 | 0.86 (0.78 - 0.96) |  | 473 | 2826 | 0.92 (0.77 - 1.1) | 0.201 |
| 60 days | No | 2877 | 14599 | Reference |  | 390 | 1943 | Reference |  |
|  | Yes | 720 | 3538 | 0.88 (0.8 - 0.97) |  | 580 | 2826 | 0.9 (0.77 - 1.05) | 0.548 |

Abbreviations: BP, blood pressure; ED: emergency department; HTN-C: hypertensive crisis; HR, hazard ratio.

^a^ Adjusted for age at ED admission, man, diabetes, hypertension, cardiovascular disease, CKD, random slope of SBP, maximum SBP, baseline eGFR, anti-platelet agents, polypharmacy.
