# Supplementary material for: Pharmacological blood pressure control and outcomes in patients with hypertensive crisis discharged from the emergency department
Source: PLoS One. 2021 Aug 17;16(8):e0251311. doi: 10.1371/journal.pone.0251311 (PMC8370605; doi:10.1371/journal.pone.0251311)
Supplement: S4 Table — (DOCX) [file pone.0251311.s004.docx]

**S4 Table.** Adjusted hazard ratios (HRs) and 95% confidence intervals of 1-year, 3-year, and 5-year cardiovascular mortality by clinical characteristics of the study population.

|  | **Pharmacological BP intervention** | **Cases** | **N** | **Adjusted HR**  **(95% CI)^a^** |  | **Cases** | **N** | **Adjusted HR**  **(95% CI)^a^** | **P for interaction** |
| --- | --- | --- | --- | --- | --- | --- | --- | --- | --- |
| **Age < 65** |  |  |  |  | **Age ≥ 65** | | | |  |
| 1-year | No | 19 | 10522 | Reference |  | 102 | 6020 | Reference |  |
|  | Yes | 11 | 3540 | 1 (0.37 - 2.67) |  | 53 | 2824 | 0.97 (0.65 - 1.44) | 0.463 |
| 3-year | No | 61 | 10522 | Reference |  | 258 | 6020 | Reference |  |
|  | Yes | 37 | 3540 | 1.04 (0.61 - 1.77) |  | 126 | 2824 | 0.93 (0.73 - 1.2) | 0.804 |
| 5-year | No | 92 | 10522 | Reference |  | 373 | 6020 | Reference |  |
|  | Yes | 56 | 3540 | 0.99 (0.63 - 1.54) |  | 182 | 2824 | 0.88 (0.71 - 1.09) | 0.536 |
| **Female** |  |  |  |  | **Male** | | | |  |
| 1-year | No | 58 | 8793 | Reference |  | 63 | 7749 | Reference |  |
|  | Yes | 36 | 3597 | 1.08 (0.65 - 1.78) |  | 28 | 2767 | 0.87 (0.5 - 1.49) | 0.449 |
| 3-year | No | 160 | 8793 | Reference |  | 159 | 7749 | Reference |  |
|  | Yes | 88 | 3597 | 0.94 (0.68 - 1.29) |  | 75 | 2767 | 0.98 (0.7 - 1.36) | 0.808 |
| 5-year | No | 232 | 8793 | Reference |  | 233 | 7749 | Reference |  |
|  | Yes | 123 | 3597 | 0.9 (0.69 - 1.17) |  | 115 | 2767 | 0.91 (0.69 - 1.2) | 0.794 |
| **Non-diabetes** | | | | | **Diabetes** | | | |  |
| 1-year | No | 93 | 14880 | Reference |  | 28 | 1662 | Reference |  |
|  | Yes | 47 | 5530 | 0.99 (0.64 - 1.55) |  | 17 | 834 | 0.93 (0.48 - 1.8) | 0.867 |
| 3-year | No | 251 | 14880 | Reference |  | 68 | 1662 | Reference |  |
|  | Yes | 117 | 5530 | 0.89 (0.68 - 1.17) |  | 46 | 834 | 1.14 (0.75 - 1.72) | 0.37 |
| 5-year | No | 380 | 14880 | Reference |  | 85 | 1662 | Reference |  |
|  | Yes | 176 | 5530 | 0.82 (0.66 - 1.03) |  | 62 | 834 | 1.17 (0.81 - 1.68) | 0.067 |
| **Non-** **hypertension** | | | | | **Hypertension** | | | |  |
| 1-year | No | 56 | 11066 | Reference |  | 65 | 5476 | Reference |  |
|  | Yes | 11 | 1516 | 0.71 (0.26 - 1.9) |  | 53 | 4848 | 1.02 (0.68 - 1.53) | 0.575 |
| 3-year | No | 132 | 11066 | Reference |  | 187 | 5476 | Reference |  |
|  | Yes | 24 | 1516 | 0.89 (0.47 - 1.68) |  | 139 | 4848 | 0.96 (0.75 - 1.23) | 0.765 |
| 5-year | No | 193 | 11066 | Reference |  | 272 | 5476 | Reference |  |
|  | Yes | 43 | 1516 | 1 (0.6 - 1.66) |  | 195 | 4848 | 0.89 (0.72 - 1.09) | 0.605 |
| **eGFR < 60** | | | | | **eGFR ≥ 60** | | | |  |
| 1-year | No | 73 | 2952 | Reference |  | 41 | 9623 | Reference |  |
|  | Yes | 44 | 1669 | 1.01 (0.64 - 1.58) |  | 17 | 3923 | 0.92 (0.48 - 1.75) | 0.859 |
| 3-year | No | 172 | 2952 | Reference |  | 112 | 9623 | Reference |  |
|  | Yes | 111 | 1669 | 1.07 (0.81 - 1.41) |  | 46 | 3923 | 0.77 (0.51 - 1.14) | 0.322 |
| 5-year | No | 245 | 2952 | Reference |  | 168 | 9623 | Reference |  |
|  | Yes | 143 | 1669 | 0.91 (0.71 - 1.15) |  | 79 | 3923 | 0.89 (0.65 - 1.23) | 0.999 |
| **Non-polypharmacy** | | | | | **Polypharmacy** | | | |  |
| 1-year | No | 66 | 11231 | Reference |  | 42 | 3130 | Reference |  |
|  | Yes | 30 | 4184 | 0.78 (0.47 - 1.31) |  | 29 | 1416 | 1.21 (0.72 - 2.05) | 0.446 |
| 3-year | No | 172 | 11231 | Reference |  | 122 | 3130 | Reference |  |
|  | Yes | 80 | 4184 | 0.88 (0.64 - 1.22) |  | 72 | 1416 | 1.03 (0.75 - 1.43) | 0.548 |
| 5-year | No | 249 | 11231 | Reference |  | 178 | 3130 | Reference |  |
|  | Yes | 122 | 4184 | 0.83 (0.63 - 1.09) |  | 97 | 1416 | 0.97 (0.74 - 1.27) | 0.73 |
| **Non-survey of end-organ damage** | | | | | **Survey of end-organ damage** | | | |  |
| 1-year | No | 27 | 5948 | Reference |  | 94 | 10594 | Reference |  |
|  | Yes | 5 | 1293 | 1.26 (0.39 - 4.06) |  | 59 | 5071 | 0.97 (0.66 - 1.43) | 0.579 |
| 3-year | No | 78 | 5948 | Reference |  | 241 | 10594 | Reference |  |
|  | Yes | 15 | 1293 | 0.96 (0.47 - 1.98) |  | 148 | 5071 | 0.95 (0.74 - 1.21) | 0.467 |
| 5-year | No | 124 | 5948 | Reference |  | 341 | 10594 | Reference |  |
|  | Yes | 30 | 1293 | 0.71 (0.39 - 1.29) |  | 208 | 5071 | 0.94 (0.76 - 1.15) | 0.175 |

Abbreviations: BP, blood pressure; ED: emergency department; HTN-C: hypertensive crisis; HR, hazard ratio.

^a^Adjusted for age at ED admission, man, diabetes, hypertension, cardiovascular disease, CKD, random slope of SBP, maximum SBP, baseline eGFR, anti-platelet agents, polypharmacy.
