# Supplementary material for: Pharmacological blood pressure control and outcomes in patients with hypertensive crisis discharged from the emergency department
Source: PLoS One. 2021 Aug 17;16(8):e0251311. doi: 10.1371/journal.pone.0251311 (PMC8370605; doi:10.1371/journal.pone.0251311)
Supplement: S5 Table — (DOCX) [file pone.0251311.s005.docx]

**S5 Table.** Adjusted hazard ratios (HRs) and 95% confidence intervals of 1-year, 3-year, and 5-year incident stroke by clinical characteristics of the study population.

|  | **Pharmacological BP intervention** | **Cases** | **N** | **Adjusted HR**  **(95% CI)^a^** |  | **Cases** | **N** | **Adjusted HR**  **(95% CI)^a^** | **P for interaction** |
| --- | --- | --- | --- | --- | --- | --- | --- | --- | --- |
| **Age < 65** |  |  |  |  | **Age ≥ 65** | | | |  |
| 1-year | No | 52 | 10120 | Reference |  | 71 | 5152 | Reference |  |
|  | Yes | 40 | 3351 | 0.81 (0.47 - 1.4) |  | 44 | 2405 | 0.84 (0.53 - 1.34) | 0.568 |
| 3-year | No | 128 | 10120 | Reference |  | 152 | 5152 | Reference |  |
|  | Yes | 81 | 3351 | 0.78 (0.54 - 1.15) |  | 85 | 2405 | 0.89 (0.65 - 1.24) | 0.533 |
| 5-year | No | 180 | 10120 | Reference |  | 209 | 5152 | Reference |  |
|  | Yes | 102 | 3351 | 0.73 (0.52 - 1.01) |  | 110 | 2405 | 0.89 (0.67 - 1.18) | 0.521 |
| **Female** |  |  |  |  | **Male** | | | |  |
| 1-year | No | 60 | 8128 | Reference |  | 63 | 7144 | Reference |  |
|  | Yes | 43 | 3265 | 0.95 (0.58 - 1.57) |  | 41 | 2491 | 0.74 (0.45 - 1.21) | 0.899 |
| 3-year | No | 136 | 8128 | Reference |  | 144 | 7144 | Reference |  |
|  | Yes | 89 | 3265 | 0.98 (0.69 - 1.37) |  | 77 | 2491 | 0.72 (0.5 - 1.04) | 0.338 |
| 5-year | No | 186 | 8128 | Reference |  | 203 | 7144 | Reference |  |
|  | Yes | 116 | 3265 | 0.92 (0.68 - 1.24) |  | 96 | 2491 | 0.71 (0.52 - 0.97) | 0.265 |
| **Non-diabetes** | | | | | **Diabetes** | | | |  |
| 1-year | No | 102 | 14007 | Reference |  | 21 | 1265 | Reference |  |
|  | Yes | 61 | 5116 | 0.64 (0.42 - 0.96) |  | 23 | 640 | 1.98 (1.02 - 3.85) | 0.05 |
| 3-year | No | 227 | 14007 | Reference |  | 53 | 1265 | Reference |  |
|  | Yes | 132 | 5116 | 0.77 (0.58 - 1.02) |  | 34 | 640 | 1.2 (0.73 - 1.95) | 0.841 |
| 5-year | No | 323 | 14007 | Reference |  | 66 | 1265 | Reference |  |
|  | Yes | 168 | 5116 | 0.72 (0.56 - 0.93) |  | 44 | 640 | 1.21 (0.78 - 1.86) | 0.492 |
| **Non-** **hypertension** | | | | | **Hypertension** | | | |  |
| 1-year | No | 60 | 10795 | Reference |  | 63 | 4477 | Reference |  |
|  | Yes | 15 | 1465 | 0.53 (0.2 - 1.42) |  | 69 | 4291 | 0.93 (0.63 - 1.36) | 0.485 |
| 3-year | No | 136 | 10795 | Reference |  | 144 | 4477 | Reference |  |
|  | Yes | 36 | 1465 | 0.83 (0.46 - 1.53) |  | 130 | 4291 | 0.85 (0.65 - 1.12) | 0.391 |
| 5-year | No | 204 | 10795 | Reference |  | 185 | 4477 | Reference |  |
|  | Yes | 39 | 1465 | 0.58 (0.32 - 1.04) |  | 173 | 4291 | 0.86 (0.68 - 1.09) | 0.688 |
| **eGFR < 60** | | | | | **eGFR ≥ 60** | | | |  |
| 1-year | No | 54 | 2492 | Reference |  | 65 | 8862 | Reference |  |
|  | Yes | 40 | 1415 | 0.97 (0.59 - 1.61) |  | 40 | 3586 | 0.71 (0.43 - 1.16) | 0.834 |
| 3-year | No | 105 | 2492 | Reference |  | 148 | 8862 | Reference |  |
|  | Yes | 64 | 1415 | 0.88 (0.6 - 1.28) |  | 85 | 3586 | 0.82 (0.59 - 1.14) | 0.23 |
| 5-year | No | 140 | 2492 | Reference |  | 203 | 8862 | Reference |  |
|  | Yes | 79 | 1415 | 0.82 (0.59 - 1.14) |  | 112 | 3586 | 0.81 (0.61 - 1.08) | 0.113 |
| **Non-polypharmacy** | | | | | **Polypharmacy** | | | |  |
| 1-year | No | 88 | 10742 | Reference |  | 26 | 2373 | Reference |  |
|  | Yes | 56 | 3939 | 0.73 (0.49 - 1.1) |  | 19 | 1075 | 1.23 (0.63 - 2.4) | 0.555 |
| 3-year | No | 180 | 10742 | Reference |  | 70 | 2373 | Reference |  |
|  | Yes | 105 | 3939 | 0.77 (0.57 - 1.04) |  | 41 | 1075 | 1.05 (0.68 - 1.62) | 0.807 |
| 5-year | No | 254 | 10742 | Reference |  | 90 | 2373 | Reference |  |
|  | Yes | 133 | 3939 | 0.7 (0.54 - 0.91) |  | 56 | 1075 | 1.13 (0.77 - 1.64) | 0.38 |
| **Non-survey of end-organ damage** | | | | | **Survey of end-organ damage** | | | |  |
| 1-year | No | 24 | 5709 | Reference |  | 99 | 9563 | Reference |  |
|  | Yes | 15 | 1229 | 1.96 (0.75 - 5.12) |  | 69 | 4527 | 0.74 (0.51 - 1.07) | 0.296 |
| 3-year | No | 81 | 5709 | Reference |  | 199 | 9563 | Reference |  |
|  | Yes | 37 | 1229 | 0.92 (0.51 - 1.67) |  | 129 | 4527 | 0.85 (0.65 - 1.12) | 0.651 |
| 5-year | No | 114 | 5709 | Reference |  | 275 | 9563 | Reference |  |
|  | Yes | 42 | 1229 | 0.85 (0.49 - 1.48) |  | 170 | 4527 | 0.82 (0.64 - 1.03) | 0.854 |

Abbreviations: BP, blood pressure; ED: emergency department; HTN-C: hypertensive crisis; HR, hazard ratio.

^a^Adjusted for age at ED admission, man, diabetes, hypertension, cardiovascular disease, CKD, random slope of SBP, maximum SBP, baseline eGFR, anti-platelet agents, polypharmacy.
