# Supplementary material for: Pharmacological blood pressure control and outcomes in patients with hypertensive crisis discharged from the emergency department
Source: PLoS One. 2021 Aug 17;16(8):e0251311. doi: 10.1371/journal.pone.0251311 (PMC8370605; doi:10.1371/journal.pone.0251311)
Supplement: S6 Table — (DOCX) [file pone.0251311.s006.docx]

**S6 Table.** Baseline demographic and clinical characteristics of the study population verified in National Health Insurance Research Database.

| **Variables** | **Missing, n (%)** | **All patients** | **Pharmacological BP intervention** | **Non-pharmacological BP intervention** | **P-value^a^** |
| --- | --- | --- | --- | --- | --- |
| **N** |  | **23601 (100)** | **6900 (29.2%)** | **16701 (70.8%)** |  |
| **Age at ED admission (year)** | 0 (0) | 60.6 (48.8, 72.8) | 63.5 (53.1, 75) | 59.4 (47, 71.8) | < 0.001 |
| **Male, n (%)** | 0 (0) | 10839 (45.9) | 3024 (43.8) | 7815 (46.8) | < 0.001 |
| **Baseline comorbidities^b^, n (%)** |  |  |  |  |  |
| Diabetes | 0 (0) | 6140 (26) | 2047 (29.7) | 4093 (24.5) | < 0.001 |
| Hypertension | 0 (0) | 14856 (62.9) | 5881 (85.2) | 8975 (53.7) | < 0.001 |
| Stroke | 0 (0) | 2974 (12.6) | 1021 (14.8) | 1953 (11.7) | < 0.001 |
| Chronic kidney disease (eGFR<60 ml/min/1.73m^2^) | 4694 (19.9) | 4909 (20.8) | 1855 (26.9) | 3054 (18.3) | < 0.001 |
| Cardiovascular disease | 0 (0) | 3944 (16.7) | 1491 (21.6) | 2453 (14.7) | < 0.001 |
| **Medication profiles^b^, n (%)** |  |  |  |  |  |
| Polypharmacy | 0 (0) | 11681 (49.5) | 3747 (54.3) | 7934 (47.5) | < 0.001 |
| Anti-platelets | 0 (0) | 6215 (26.3) | 2180 (31.6) | 4035 (24.2) | < 0.001 |
| **Outcome, n (%)** |  |  |  |  |  |
| **ED re-visit or inpatient service, n (%)** |  |  |  |  |  |
| 7-day | 0 (0) | 2711 (11.5) | 830 (12) | 1881 (11.3) | 0.093 |
| 30-day | 0 (0) | 4746 (20.1) | 1441 (20.9) | 3305 (19.8) | 0.056 |
| 60-day | 0 (0) | 5953 (25.2) | 1810 (26.2) | 4143 (24.8) | 0.022 |
| **Incident stroke (after index date)**^c^ | 0 (0) | 3391 (16.4) | 1123 (19.1) | 2268 (15.4) | < 0.001 |
| **Cardiovascular mortality (after index date)** | 0 (0) | 3022 (12.8) | 955 (13.8) | 2067 (12.4) | 0.002 |

Although the number of study population we analyzed using the CMUH-CRDR (N = 22,906) was 695 patients less than the population we analyzed using the NHIRD data, both population came from the same source population. In addition, the age and gender distribution was comparable (age:60.2 vs 60.6; male:43.5% vs 46.8%). The discrepancy of the number of patients was caused by the different data source and variable format in CMUH-CRDR and in NHIRD.

Abbreviations: CMUH-CRDR, China Medical University Hospital-Clinical Research Data Repository; ED, emergency department; eGFR indicates estimated glomerular ﬁltration rate; HTN, hypertension; IQR, interquartile range.

^a^ P-values are calculated by Kruskal-Wallis test for continuous variables and Chi-square test (or Fisher’s exact test as appropriate) for categorical variables.

^b^ Baseline comorbidities/ medication profiles that were diagnosed/ taken within 1 year prior to the index date.

^c^ The outcome of incident stroke exclude patients who had ever stroke before index date 1 years, and 20627 patients were left for analysis.

There are 20627 patients overall, 5879 (28.5%) and 14748 (71.5%) patients in with anti-HTN medications group and without anti-HTN medications group respectively.
