# Supplementary material for: Pharmacological blood pressure control and outcomes in patients with hypertensive crisis discharged from the emergency department
Source: PLoS One. 2021 Aug 17;16(8):e0251311. doi: 10.1371/journal.pone.0251311 (PMC8370605; doi:10.1371/journal.pone.0251311)
Supplement: S7 Table — BP, blood pressure; ED, emergency department. (DOCX) [file pone.0251311.s007.docx]

**S7 Table.** Hazard ratios (HRs) with 95% confidence interval for ED revisit or inpatient admission, incident stroke, and cardiovascular mortality according to the exposure of pharmacological BP control in the ED setting verified using National Health Insurance Research Database. BP, blood pressure; ED, emergency department.

| **Pharmacologically blood pressure reduction** |  |  |  |  | **Crude HR**  **(95% CI)** | **Model 1^b^** | **Model 2^c^** | **Model 3^d^** |
| --- | --- | --- | --- | --- | --- | --- | --- | --- |
|  | **N** | **Case** | **Person-**  **year** | **Incidence^a^** |  | **Adjusted HR**  **(95% CI)** | **Adjusted HR**  **(95% CI)** | **Adjusted HR**  **(95% CI)** |
| **ED revisit or inpatient service** | |  |  |  |  |  |  |  |
| 7-day |  |  |  |  |  |  |  |  |
| No | 16701 | 1881 | 299.474 | 6281.0 | 1.00 (Ref) | 1.00 (Ref) | 1.00 (Ref) | 1.00 (Ref) |
| Yes | 6900 | 830 | 123.379 | 6727.2 | 1.07 (0.99 - 1.16) | 0.95 (0.87 - 1.03) | 0.91 (0.83 - 1) | 0.92 (0.84 - 1.01) |
| 30-day |  |  |  |  |  |  |  |  |
| No | 16701 | 3305 | 1182.215 | 2795.6 | 1.00 (Ref) | 1.00 (Ref) | 1.00 (Ref) | 1.00 (Ref) |
| Yes | 6900 | 1441 | 483.532 | 2980.2 | 1.06 (1 - 1.13) | 0.93 (0.87 - 1) | 0.91 (0.85 - 0.97) | 0.92 (0.86 - 0.98) |
| 60-day |  |  |  |  |  |  |  |  |
| No | 16701 | 4143 | 2246.305 | 1844.4 | 1.00 (Ref) | 1.00 (Ref) | 1.00 (Ref) | 1.00 (Ref) |
| Yes | 6900 | 1810 | 915.765 | 1976.5 | 1.07 (1.01 - 1.13) | 0.94 (0.88 - 0.99) | 0.91 (0.85 - 0.97) | 0.92 (0.87 - 0.98) |
| **Cardiovascular mortality** |  |  |  |  |  |  |  |  |
| 1-year |  |  |  |  |  |  |  |  |
| No | 16701 | 609 | 16362.637 | 37.2 | 1.00 (Ref) | 1.00 (Ref) | 1.00 (Ref) | 1.00 (Ref) |
| Yes | 6900 | 306 | 6740.683 | 45.4 | 1.22 (1.06 - 1.4) | 0.99 (0.86 - 1.14) | 0.92 (0.79 - 1.07) | 0.92 (0.79 - 1.07) |
| 3-year |  |  |  |  |  |  |  |  |
| No | 16701 | 1411 | 47692.226 | 29.6 | 1.00 (Ref) | 1.00 (Ref) | 1.00 (Ref) | 1.00 (Ref) |
| Yes | 6900 | 673 | 19542.53 | 34.4 | 1.16 (1.06 - 1.27) | 0.95 (0.86 - 1.04) | 0.83 (0.75 - 0.92) | 0.83 (0.75 - 0.93) |
| 5-year |  |  |  |  |  |  |  |  |
| No | 16701 | 1906 | 77734.373 | 24.5 | 1.00 (Ref) | 1.00 (Ref) | 1.00 (Ref) | 1.00 (Ref) |
| Yes | 6900 | 892 | 31756.296 | 28.1 | 1.14 (1.06 - 1.24) | 0.91 (0.84 - 0.99) | 0.78 (0.71 - 0.85) | 0.78 (0.72 - 0.86) |
| **Incident stroke** |  |  |  |  |  |  |  |  |
| 1-year |  |  |  |  |  |  |  |  |
| No | 14748 | 736 | 14271.1 | 51.6 | 1.00 (Ref) | 1.00 (Ref) | 1.00 (Ref) | 1.00 (Ref) |
| Yes | 5879 | 406 | 5612.88 | 72.3 | 1.4 (1.24 - 1.58) | 1.04 (0.92 - 1.19) | 0.99 (0.86 - 1.14) | 1 (0.87 - 1.14) |
| 3-year |  |  |  |  |  |  |  |  |
| No | 14748 | 1506 | 41469.65 | 36.3 | 1.00 (Ref) | 1.00 (Ref) | 1.00 (Ref) | 1.00 (Ref) |
| Yes | 5879 | 803 | 16136.16 | 49.8 | 1.36 (1.25 - 1.49) | 1.03 (0.94 - 1.13) | 0.97 (0.88 - 1.07) | 0.97 (0.88 - 1.07) |
| 5-year |  |  |  |  |  |  |  |  |
| No | 14748 | 2126 | 67277.33 | 31.6 | 1.00 (Ref) | 1.00 (Ref) | 1.00 (Ref) | 1.00 (Ref) |
| Yes | 5879 | 1074 | 25997.65 | 41.3 | 1.3 (1.21 - 1.4) | 1.01 (0.94 - 1.1) | 0.93 (0.85 - 1.01) | 0.93 (0.86 - 1.02) |

^a^ Incidence = No. of cases / person-years*1000.

^b^ Model 1: Adjusted for age at ED admission, gender, diabetes, hypertension, cardiovascular disease, chronic kidney disease.

^c^ Model 2: Further adjusted for random slope of systolic blood pressure, maximum systolic blood pressure, baseline estimated glomerular ﬁltration rate.

^d^ Model 3: Further adjusted for anti-platelet agents, polypharmacy.
