# Supplementary material for: Pharmacological blood pressure control and outcomes in patients with hypertensive crisis discharged from the emergency department
Source: PLoS One. 2021 Aug 17;16(8):e0251311. doi: 10.1371/journal.pone.0251311 (PMC8370605; doi:10.1371/journal.pone.0251311)
Supplement: S9 Table — BP, blood pressure; ED, emergency department. (DOCX) [file pone.0251311.s009.docx]

**S9 Table.** E-value for point estimate and confidence interval for ED revisit or inpatient admission, incident stroke, and cardiovascular mortality according to the exposure of pharmacological BP control in the ED setting. BP, blood pressure; ED, emergency department.

| **Pharmacologically blood pressure reduction** | **E-value for point estimate** | **E-value for confidence interval** |
| --- | --- | --- |
| **ED revisit or inpatient service** | |  |
| 7-day | 1.32 | 1 |
| 30-day | 1.39 | 1.17 |
| 60-day | 1.39 | 1.2 |
| **Cardiovascular mortality** | |  |
| 1-year | 1.21 | 1 |
| 3-year | 1.29 | 1 |
| 5-year | 1.5 | 1 |
| **Incident stroke** | |  |
| 1-year | 1.67 | 1 |
| 3-year | 1.67 | 1 |
| 5-year | 1.77 | 1 |
