# Supplementary material for: Pharmacological blood pressure control and outcomes in patients with hypertensive crisis discharged from the emergency department
Source: PLoS One. 2021 Aug 17;16(8):e0251311. doi: 10.1371/journal.pone.0251311 (PMC8370605; doi:10.1371/journal.pone.0251311)
Supplement: S10 Table — BP, blood pressure; ED, emergency department. (DOCX) [file pone.0251311.s010.docx]

**S10 Table.** Hazard ratios (HRs) with 95% confidence interval for ED revisit or inpatient admission, incident stroke, and cardiovascular mortality according to the exposure of pharmacological BP control in the ED setting based on multiple imputation data. BP, blood pressure; ED, emergency department.

| **Pharmacologically blood pressure reduction** | **Original Model 3^a^** | **Model 3 with multiple imputations** |
| --- | --- | --- |
|  | **Adjusted HR (95% CI)** | **Adjusted HR (95% CI)** |
| **ED revisit or inpatient service** | | |
| 7-day |  |  |
| No | 1.00 (Ref) | 1.00 (Ref) |
| Yes | 0.94 (0.84 - 1.06) | 0.96 (0.86 - 1.08) |
| 30-day |  |  |
| No | 1.00 (Ref) | 1.00 (Ref) |
| Yes | 0.89 (0.82 - 0.97) | 0.92 (0.84 - 1.00) |
| 60-day |  |  |
| No | 1.00 (Ref) | 1.00 (Ref) |
| Yes | 0.89 (0.82 - 0.96) | 0.92 (0.85 - 0.99) |
| **Cardiovascular mortality** | | |
| 1-year |  |  |
| No | 1.00 (Ref) | 1.00 (Ref) |
| Yes | 0.97 (0.67 - 1.41) | 1.04 (0.74 - 1.47) |
| 3-year |  |  |
| No | 1.00 (Ref) | 1.00 (Ref) |
| Yes | 0.95 (0.75 - 1.19) | 0.93 (0.75 - 1.16) |
| 5-year |  |  |
| No | 1.00 (Ref) | 1.00 (Ref) |
| Yes | 0.89 (0.74 - 1.08) | 0.93 (0.78 - 1.11) |
| **Incident stroke** | | |
| 1-year |  |  |
| No | 1.00 (Ref) | 1.00 (Ref) |
| Yes | 0.84 (0.59 - 1.19) | 0.92 (0.66 - 1.28) |
| 3-year |  |  |
| No | 1.00 (Ref) | 1.00 (Ref) |
| Yes | 0.84 (0.66 - 1.08) | 0.93 (0.74 - 1.16) |
| 5-year |  |  |
| No | 1.00 (Ref) | 1.00 (Ref) |
| Yes | 0.81 (0.65 - 1.01) | 0.86 (0.7 - 1.04) |

^a^Model 3: Adjusted for age at ED admission, gender, diabetes, hypertension, cardiovascular disease, chronic kidney disease, random slope of systolic blood pressure, maximum systolic blood pressure, baseline estimated glomerular ﬁltration rate, anti-platelet agents, polypharmacy.
